# The effect of inhalation of *Citrus sinensis* flowers and *Mentha spicata* leave essential oils on lung function and exercise performance: a quasi-experimental uncontrolled before-and-after study

**DOI:** 10.1186/s12970-016-0146-7

**Published:** 2016-09-22

**Authors:** Nidal Amin Jaradat, Hamzeh Al Zabadi, Belal Rahhal, Azmi Mahmoud Ali Hussein, Jamal Shaker Mahmoud, Basel Mansour, Ahmad Ibrahim Khasati, Abdelkhaleq Issa

**Affiliations:** 1 Department of Pharmacy, Faculty of Medicine and Health Sciences, An-Najah National University, P.O. Box 7, Nablus, Palestine; 2 Public Health Division, Faculty of Medicine and Health Sciences, An-Najah National University, P.O. Box 7, Nablus, Palestine; 3 Physiology, Pharmacology and Toxicology Division, Faculty of Medicine and Health Sciences, An-Najah National University, P.O. Box 7, Nablus, Palestine; 4 Faculty of Physical Education, An-Najah National University, P.O. Box 7, Nablus, Palestine; 5 Faculty of Law, An-Najah National University, P.O. Box 7, Nablus, Palestine; 6 Department of Chemistry, Arab American University of Jenin, Jenin, Palestine; 7 Faculty of Humanities, An-Najah National University, P.O. Box 7, Nablus, Palestine

**Keywords:** *Citrus sinensis*, *Mentha spicata*, Essential oil, Lung function, Athletic performance

## Abstract

**Background:**

Recently, there has been an increased interest in the effects of essential oils on athletic performances and other physiological effects. This study aimed to assess the effects of *Citrus sinensis* flower and *Mentha spicata* leaves essential oils inhalation in two different groups of athlete male students on their exercise performance and lung function.

**Methods:**

Twenty physical education students volunteered to participate in the study. The subjects were randomly assigned into two groups: *Mentha spicata* and *Citrus sinensis* (ten participants each). One group was nebulized by *Citrus sinensis* flower oil and the other by *Mentha spicata* leaves oil in a concentration of (0.02 ml/kg of body mass) which was mixed with 2 ml of normal saline for 5 min before a 1500 m running tests. Lung function tests were measured using a spirometer for each student pre and post nebulization giving the same running distance pre and post oils inhalation.

**Results:**

A lung function tests showed an improvement on the lung status for the students after inhaling of the oils. Interestingly, there was a significant increase in Forced Expiratory Volume in the first second and Forced Vital Capacity after inhalation for the both oils. Moreover significant reductions in the means of the running time were observed among these two groups. The normal spirometry results were 50 %, while after inhalation with *M. spicata* oil the ratio were 60 %.

**Conclusion:**

Our findings support the effectiveness of *M. spicata* and *C. sinensis* essential oils on the exercise performance and respiratory function parameters. However, our conclusion and generalisability of our results should be interpreted with caution due to small sample size and lack of control groups, randomization or masking. We recommend further investigations to explain the mechanism of actions for these two essential oils on exercise performance and respiratory parameters.

**Trial registration:**

ISRCTN10133422, Registered: May 3, 2016.

## Background

In recent times, the world has witnessed an increase in the use of essential oils to treat diseases and to promote better health [1, 2]. The natural plant’s essential oils are widely used in medicine and pharmacy for many purposes due to their evidence based physiological and psychological effects [3, 4]. They are also considered one of the most important branches of Complementary and Alternative Medicine, which is called aromatherapy that focuses on the usage of essential oils for the treatment of various illnesses by using natural essential oils [5–8].

Essential oils can cleanse cellular receptor sites of medications, petrochemicals and other disruptors of intercellular communication and can chelate heavy metals and other toxins, helping to remove and flush them through the kidneys, lungs, sweat, colon and liver. In fact, they increase the body’s ability to absorb nutrients and vitamins [9–11].

Moreover, many of the essential oils are used in the pharmaceutical industry as an active ingredient in the pharmaceutical formulations such as Eucalyptus, Peppermint, Thyme, Anise, Fennel oils and many others or they are used in the pharmaceutical industry as excipients as well as most of the flavoring agents which are used to improve the odor and taste of drugs that are isolated from various plants containing essential oils [12–15].

In the last thirty years, a series of studies were conducted to evaluate the effects of essential oils smelling on the behaviors, creativity, mood and many other psychological and physiological effects [16–20]. In vapor forms in the folk medicine *Citrus sinensis* flower oil used as sedative and *Mentha spicata* leaves oil used as bronchodilator, for that our study aimed to investigate their effects on the lung function and to evaluate their effects on athletic performance [21, 22].

*Citrus sinensis* (L.), orange or sweet orange is a small tree in the Rutaceae family that originated in southern China and now cultivated worldwide in tropical, semi-tropical, and some warm temperate regions, and have become the most widely planted fruit tree in the world. The fragrant white flowers, produced singly or in cluster of up to 6 flowers, which are around 5 cm wide, with 5 petals and 20 to 25 yellow stamens [23]. The flowers oil consist mainly from sabinene, linalool, limonene and trans-nerolidol [24] and are used as an antimicrobial, stomachic, carminative and flavoring agent as well as orange flowers water used in Palestine as food [25, 26].

*Mentha spicata* L.(Spearmint) belongs to the family Lamiaceae and characterized by its leaves essential oil that is of great economic importance and being used widely in cosmetic, pharmaceutical and food industries [27–29]. The major constituents of *M. spicata* leaves essential oil are carvone, limonene, dihydrocarvone, 1,8-cineol, β-bourbonene, β-caryophyllene, myrcene and α-pinene and aromatherapists. This oil is used for its antispasmodic, local anesthetic, astringent, carminative, decongestant, digestive, diuretic and expectorant effects [30–32].

Lung function tests (LFTs) are usually measured using the spirometer [33]. The spirometer is used to differentiate between obstructive and restrictive diseases and assess the degree of associated changes [34, 35]. Such parameters include Forced Expiratory Volume in the first second (FEV1) and Forced Vital Capacity (FVC). The specificity and sensitivity of spirometry in the diagnosis of obstructive lung disease are reported as 84 and 92 %, respectively [36]. The FEV1 is the maximum air volume is exhaled with maximal effort in the first second from a position of full inspiration. The FEV1/FVC ratio is reduced in obstructive patterns, but it is normal or increased in restrictive patterns as both nominator and denominator proportionally change [37].

In the last thirty years some studies have been conducted to evaluate the odors and consumption of essential oils on physical and psychological activities. Most of these studies focused on peppermint plant but unfortunately and according to the best of authors’ knowledge there were no previous studies about *M. spicata* and *C. sinensis* oils inhalations on the lung function and/or the athletic performance.

## Methods

### Instrumentations

During this study, the following instruments were used: ultrasonic-microwave cooperative extractor/reactor (CW-2000, China), balance (Radw ag, AS 220/c/2, Poland), Philips respironics inhalators nebulizers (75644321, China) and Care-fusion spirometer (ME44QY 08563848, UK).

### Collection and preparing plant materials

The leaves of *M. spicata* and *C. sinensis* flowers were collected during its flowering time from Tulkarem region (Palestine) during April, 2015. Botanical identification was carried out by Pharmacognosist Dr. Nidal Jaradat from the Pharmacognosy and Herbal Products Laboratory, Faculty of Medicine and Health Sciences, An-Najah National University, Nablus. The identification process was conducted using live herbal specimens and photographs from books. Voucher specimens were deposited in the Pharmacognosy and Herbal Products Laboratory under the code numbers: Pharm-PCT-2774 for *M. spicata* and was Pharm-PCT-2775 for *C. sinensis.*

To extract volatile oil, the leaves of *M. spicata* and the flowers *C. sinensis* were separated carefully and then washed twice with distilled water.

### Essential oils extraction

The essential oil of *M. spicata* leaves were extracted using a microwave oven as described by Jaradat, 2016 with some modifications [38]. The power of the microwave oven was set at 1000 W. Clevenger apparatus with a 1 L round-bottom flask containing 100 g of *M. spicata* leaves was placed inside the microwave oven. About 500 ml distilled water was then added into the flask containing the powder. The flask was then connected to Clevenger apparatus. Microwave distillation was carried out three times for 15 min each at 100 °C. The obtained volatile oil was collected into a clean beaker and chemically dried then the purified essential oil was weighed and stored in tightly-closed amber-colored bottles at −4 °C in the refrigerator. The same procedure was repeated for extraction of *C. sinensis* essential oil flowers.

### Subjects and study design

Twenty male university students from the faculty of physical education at An-Najah National University in Nablus-Palestine volunteered and were randomly assigned into two different groups (10 participants each) to take part in the experiments using a non-randomized quasi-experimental uncontrolled before-and-after study design for each group. The study was single blind at the level of participants. One group (10 students) was nebulized with *M. spicata* oil, while the other group (10 students) was nebulized with *C. sinensis* flowers oil (0.02 ml/kg of body mass of each oil) mixed with 2 ml of normal saline 5 min before a 1500 m running test according to the method validated by Spencer and Gastin, 2001. Traditionally, it was thought that in the middle distance track running the relative energy system was 75 % aerobic and 22 % anaerobic and a 2–3 % of the energy coming from the creatine-phosphate system. But recently, the aerobic energy system contributes significantly to the energy supply during middle distance running and long sprint event. Moreover, the relative anaerobic and aerobic system contributions of the four events (200 m, 400 m, 800 m, and 1500 m). In the middle distance running such as 1500 m event depends on the aerobic 84 % and anaerobic energy system which is greater than it was traditionally thought, so during this event the need of oxygen increases with the event duration from 200 to 1500 m running race [39].

Moreover, the 1500 m distance was required to ensure the duration of effect of the oils (if any) as inhaled route of administration and the greater distances could have been taken into consideration if the interventions were given orally or by another route of administration, however, our estimation was that this distance could be representable of such route of administration given at the same time that the same group conducted the running pre and post intervention.

A washout period of three days (from Sunday to Wednesday) between pre and after test for each group was given to rule out any fatigue or significant impact on our results pre and post test. Lung function tests were measured using a spirometer for each student pre and post nebulization. The study was carried out in May, 2016.

### Methodology

The subjects were familiarized with the spirometry. Lung function tests were measured using a spirometer for each student pre and post inhalations with *M. spicata* and *C. sinensis* oils. The specificity and sensitivity of spirometer in diagnosis of obstructive lung disease are reported as 84 and 92 %, respectively [40]. While in the diagnosis of restrictive lung disease, it has a sensitivity and specificity of 42.2 and 94.3 %, respectively [41]. FEV1 is the maximum air volume exhaled with maximal effort in the first second from a position of full inspiration. This value declines less severely with restrictive diseases than obstructive diseases. FVC, on the other hand, is the maximal air volume exhaled with maximal effort from a position of full inspiration [42], and is reduced by an airflow obstruction and ventilation restriction, which results from lung-exterior factors such as skeletal pains or intrinsic lung disease, especially restrictive ones [43]. In the latter, there is a decline in the lung compliance associated with the presence of partial or diffuse lung fibrosis. These fibrotic changes render the lung smaller and stiffer, leading to a decrease in the FVC. The FEV1/FVC ratio is reduced in obstructive patterns, but it is normal or even increased in restrictive patterns as both nominator and denominator proportionally change.

### Data collection form

Different sections were included. The first was the demographic section, which contained questions regarding age, gender, education level, health history, smoking status and body mass index. Then, spirometry was performed using a Spirometer apparatus. Regular guidelines for spirometer testing were followed [44]. The subjects were seated during the test, with the nose clipped to prevent air leakage through the nares. Three attempts for each subject were allowed, and the best Spirograph was selected automatically by the spirometer. Forced spirometry was measured for each subject. For each subject, FEV1, FVC, FEV1/FVC ratios were assessed.

### Data analysis

All data has been entered and analyzed using the statistical software package SPSS (Statistical Package for the Social Sciences) version 16. The main study outcomes were tested for normality using Shapiro-Wilk significant test and found not to be normally distributed. Qualitative variables have been expressed as frequency tables and bar charts. The non-parametric Two-related samples Wilcoxon test was used to test for the differences in the means between pre- and post-inhalers of the continuous outcomes. *P-*value of less than 0.05 was always considered statistically significant.

## Results

### Socio-demographic characteristics

All participants were single. The mean age (standard deviation; SD) of *M. spicata* group participants was 19.10 (1.45) years and 50 % of them were smoking 1–10 cigarettes per day. In the *C. sinensis* group, however, the mean age (SD) was 19.80 (1.22) years and the vast majority (90 %) were non-smokers. Table 1 shows the socio-demographic characteristics details of the study participants for each group.

**Table 1:** Socio-demographic characteristics of the study population given *M. spicata* and *C. sinensis* oils (*N* = 10 for each group)

Variable	*M. spicata* oil group	*C. sinensis* oil group
	n (%)^a^	
Gender		
-Male	10 (100)	10 (100)
Marital status		
-Single	10 (100)	10 (100)
Smoking status (cigarette/day)		
-Non-smokers	4 (40)	9 (90)
-1-10	5 (50)	1 (10)
-11-20	1 (10)	0 (0)
Age (year)	19.10 ± 1.45^b^	19.80 ± 1.22^b^
Weight (kg)	70.30 ± 5.33^b^	73.10 ± 8.77^b^
Height (meter; m)	1.77 ± 0.038^b^	1.78 ± 0.056^b^
BMI (kg/m^2^)	22.49 ± 2.19^b^	22.90 ± 2.93^b^

^a^Data is presented as frequency (percent) (*N* = 10). ^b^Mean ± standard deviation

### Respiratory parameters assessment

**Fig. 1:**
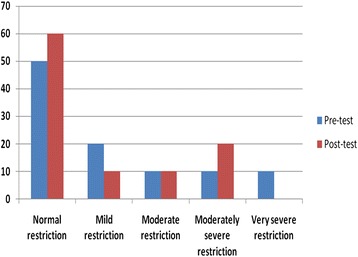
Respiratory parameters for participants before and after inhalation of *M. spicata* oil

**Fig. 2:**
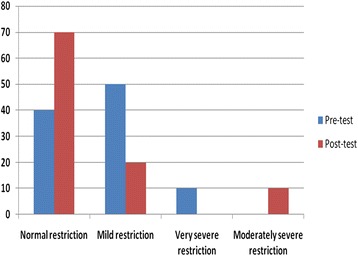
Respiratory parameters for participants before and after oil inhalation of *C. sinensis* oil

A lung function test showed an improvement on the lung status for the students after their inhalation of the oils. Figure 1 showed that the normal spirometry results were 50 %, while after inhalation with *M. spicata* oil the ratio were 60 %. On the other hand, there were a more increase in normal lung status for students who inhaled *C. sinensis* oil (40 % before and 70 % after; Fig. 2).

Interestingly, Table 2 shows that there is a significant increase in FEV1 and FVC in the post-test students after their inhalation of the *M. spicata* and *C. sinensis* oils.

**Table 2:** Respiratory parameters in the post- and pre-inhalers measurements for the study outcomes of the participants given *M. spicata* (*n* = 10) and *C. sinensis* oils (*n* = 10) when running equal distance of 1500 m before and after

Outcome variable	*M. spicata* oil group			*C. sinensis* oil group		
	Mean ± SD^a^	Mean difference ± SD	*P*-value*	Mean ± SD^a^	Mean difference ± SD	*P*-value*
FEV1						
- Post-inhaler	4.47 ± 0.23			4.66 ± 0.33		
- Pre-inhalers	3.72 ± 1.00	0.75 ± 1.16	**0.074** ***	3.83 ± 1.39	0.83 ± 1.50	**0.037** **
FVC						
- Post-inhaler	5.32 ± 0.31			5.57 ± 0.44		
- Pre-inhalers	4.41 ± 0.58	0.91 ± 0.75	**0.007** **	3.99 ± 1.45	1.57 ± 1.59	**0.005** **
FEV1/FVC						
- Post-inhaler	84.10 ± 0.31			84.10 ± 0.31		
- Pre-inhalers	85.00 ± 20.43	−0.90 ± 20.35	0.574	94.10 ± 8.58	−1.00 ± 8.56	**0.021** **

*P-value for the mean differences of the Two-related samples Wilcoxon test. ** Significance *P*-values (less than 0.05), *** A borderline significance

^a^SD, standard deviation; FEV1, Forced Expiratory Volume in the first second; FVC, Forced Vital Capacity. FEV1/FVC, Ratio of FEV1 over FVC

The FEV1/FVC ratios were not significant in *M. spicata* oil participant while there is a significant decrease toward the normal in *C. sinensis* oil participants.

Furthermore, there was a statistically significant reduction in the mean running time after inhalation of *M. spicata* oil and *C. sinensis* oil among the study participants (*p*-values, 0.007 and 0.005; respectively) as shown in Table 3.

**Table 3:** Changes in exercise performance (time in seconds) for the participants given *M. spicata* (*n* = 10) and *C. sinensis* oils (*n* = 10) when running equal distance of 1500 m before and after oil inhalations

Outcome variable	*M. spicata* oil group			*C. sinensis* oil group		
	Mean ± SD^a^	Mean difference ± SD	*P*-value*	Mean ± SD^a^	Mean difference ± SD	*P*-value*
Time (sec)^b^	300.50 ± 19.89			294.70 ± 14.20		
- Post-inhaler	340.70 ± 16.08			347.90 ± 15.59	-	
- Pre-inhalers		−40.2 ± 2.32	**0.007** **		53.20 ± 7.94	**0.005** **

*P-value for the mean differences of the Two-related samples Wilcoxon test. ** Significant *P*-values (less than 0.05)

^a^SD, standard deviation

^b^Data in the table is expressed as seconds

## Discussion

Our findings showed that the essential oils isolated from *M. spicata* leaves and *C. sinensis* flowers enhanced athletic performance and lung function. Results showed that there is a significant increase in FEV1 and FVC in the post-test students after their inhalation of the *M. spicata* and *C. sinensis* oils. Furthermore, the running times for each group were significantly decreased for both *M. spicata* and *C. sinensis* nebulized groups (*p*-values, 0.007 and 0.005; respectively).

A scientific Study which was conducted by Saeki and Tanaka, 2005 had approved that an inhaling fragrances had affected the relieving of pricking pain sensation and suppressed autonomic responses and they suggested that aromatherapy may have more palliative effect on chronic rather than pricking pain [45].

Studies have shown that inhalations of various species of peppermint were effective in reducing muscle pain and fatigue as well as they had muscle relaxation effect [46–48].

Another investigation was conducted by McKenzie and Hedge, 2005 [49] about the effects of inhalation of peppermint oil on running performance under different conditions. Eighteen young female subjects run 3.25 miles and were divided into groups; wearied a peppermint scented mask group, and unscented mask group. The results showed that peppermint inhalation had significantly lower heart rates during the running task.

A study of Dedeçay’s, 1995 [50] showed that the aqueous solution containing rosemary and peppermint which was given to French cyclists made muscle relaxation and decreased muscular fatigues. Further to this, studies on peppermint inhalation had approved that this plant essential oil reduced the perceived efforts, temporal workload, physical workload and frustration [51–53].

Another study was conducted by Asghar S., 2011, on the effects of peppermint inhalation on VO_2_ max and reaction time, on 20 male athletes voluntarily participated in the study and the results showed that there is a meaningful relationship between the inhalation of peppermint with aerobic performance and reaction time [54].

Results of the current study, confirmed with the findings of Meamarbashi and Rajabi, 2013 and Raudenbush et al., 2001, who examined the effects of the administration of peppermint on the performance of athletes during exercises [51, 55].

Natural plants essential oils have been traditionally used in the treatment of various physiological and psychological disorders. The use of essential oils in medicine began in the ancient Egyptian Era, and has continued ever since [56, 57]. One of the most popular parts of complementary and alternative medicine is considered to be aromatherapy, which depends only on essential oils utilization for treatment of various diseases and this branch has spread worldwide, despite the lack of scientific basis for the effectiveness of essential oils [58]. On the other hand, the long history of essential oils usages in medicine and pharmacy suggests that they may indeed be effective. The odor of essential oils is believed to be important for their effectiveness in treating various illnesses [59].

Most of the essential oils are considered to be safe and their safety had been monitored in different ways, as well as these essential oils had been used from the ancient times in perfumery, cosmetics and food industries [60]. Most of these studies focused on peppermint plant but unfortunately and according to the best of authors’ knowledge there were no previous studies about *M. spicata* and *C. sinensis* oils inhalations on the lung function and on the athletic performance.

Future study is needed to demonstrate oral supplementation of *M. spicata* and *C. sinensis* essential oils to improve athletic performance and post-exercise recovery; another future objective would be to clarify their mechanism of action and to explain their physiological and pharmacological effects.

### Study limitations

This study could have some limitations. The absent of control groups is one of the limitations. However, no control groups have been proposed in order to ensure that the intervention reaches the maximum number of students agreed to participate. In addition, in the design could also be another source of limitations in this study. Indeed, we conduct a non-randomized quasi-experimental uncontrolled before-and-after study. However, we are aware that in this design secular trends or sudden changes make it difficult to attribute observed changes to the intervention. Further limitations of this design could be that the occurrence of over-estimation of the effects of intervention. Interestingly enough, by using this design we were able to measure performance before and after the introduction of intervention in the same study group and the observed differences in performance were assumed to be due to the intervention. This study design has the advantages of that it is relatively simple to conduct and participants are having same characteristics pre-and post test and it is a design superior to observational studies. Furthermore, due to lack of resources and difficulty of circumstances in our country and university we found this the most robust design possible to minimize bias and maximize generalisability.

Regarding the small sample size which could have affected the study statistical power, we are fully aware of this limitation but we were not be able to recruit more due to voluntary participation. Therefore, we found it fairly enough to divide the 20 students ten by ten in each group in order to run this study. We also did not conduct between group changes as we did not aim to perform comparison between the two groups with the different oils intervention but we only aimed to assess the outcome for each intervention in the same group pre-and post test.

As we have indicated above, we used a quasi-experimental uncontrolled before-and-after study design and we did not aim to compare between groups, therefore, differences in smoking or any other variations between groups were not taken into consideration in the analysis as we have measured the changes for each intervention separately among the same group pre- and post test.

There was no dietary control in this study. However, we believe that those are informative groups of students who might have same weekly dietary intake level and we asked them to restrict any energy stimulator intakes during the week of the study. Therefore, dietary intake could have minimal effects on our findings.

## Conclusions

To our knowledge, this is the first study that explored the effect of inhalation of *M. spicata* and *C. sinensis* essential oils on exercise performance and lung function. Our findings support the effectiveness of *M. spicata* and *C. sinensis* essential oils on the exercise performance and respiratory function parameters. However, our conclusion and generalisability of our results should be interpreted with caution due to small sample size and lack of control groups, randomization or masking. No control groups have been proposed in order to ensure that the intervention reaches the maximum number of students agreed to participate. We recommend further investigations to explain the mechanism of actions for these two essential oils on exercise performance and respiratory gas exchange parameters. Differences in running distance, duration of study and inhalation of two different unstudied before essential oils instead of inhalation of peppermint aroma could be the important characteristics of this study compared to previous researches.

## Abbreviations

*C. sinensis*: *Citrus sinensis*
FEV1: Forced Expiratory Volume in the first second
FVC: Forced vital capacity
*M. spicata*: *Mentha spicata*
